# Association between neutrophil percentage-to-albumin ratio and chronic subdural hematoma: a retrospective case-control study

**DOI:** 10.3389/fneur.2026.1831131

**Published:** 2026-07-21

**Authors:** Guanda Han, Yifan Lv, Mingjie Wang, Tao Sun, Xiaohui Dong, Mingliang Deng, Hongjie Zhai, Hui Zhang, Lei Li

**Affiliations:** Department of Neurosurgery at the First Affiliated Hospital of Bengbu Medical University, Bengbu, China

**Keywords:** association analysis, chronic subdural hematoma, inflammation-nutrition indicator, neutrophil percentage-to-albumin ratio, risk factors

## Abstract

**Objective:**

To investigate the association between neutrophil percentage-to-albumin ratio (NPAR) and chronic subdural hematoma (CSDH), and evaluate its discriminative efficacy.

**Methods:**

Clinical data of 87 CSDH patients and 130 healthy controls (2023–2025) were retrospectively analyzed. Logistic regression, ROC curves, bootstrap validation (1,000 iterations), VIF, and Hosmer-Lemeshow test were performed. Stratified analyses by age (<70 vs. ≥70 years) and sex assessed association robustness.

**Results:**

NPAR was significantly higher in CSDH patients (15.31 ± 2.71 vs. 12.47 ± 1.97, *P* < 0.001). Multivariate analysis showed NPAR (OR = 1.88, 95% CI: 1.41–2.51, *P* < 0.001), age, male sex, and lipids were independently associated with CSDH. NPAR demonstrated optimal discriminative efficacy (AUC = 0.808, 95% CI: 0.747–0.868; cutoff 14.18; sensitivity 71.3%; specificity 83.8%). Bootstrap-validated multivariate AUC was 0.914 (optimism-corrected 0.907). In age-balanced strata, NPAR remained significantly higher in CSDH (male <70 years: *P* < 0.001; female ≥70 years: *P* < 0.05). Progressive adjustment attenuated the NPAR OR by only 9.6% (from 1.768 to 1.605), with full adjustment yielding OR = 1.626 (95% CI: 1.238–2.135). VIF < 5; Hosmer-Lemeshow *P* = 0.072.

**Conclusion:**

NPAR showed a significant independent association with CSDH. As a simple, economical inflammation-nutrition composite indicator, it may aid CSDH risk assessment. However, this association cannot be directly extrapolated as a clinically operational diagnostic threshold, warranting validation in target screening populations.

## 1 Introduction

Chronic subdural hematoma (CSDH) is a chronic hematoma occurring between the dura mater and arachnoid membrane, encapsulated by a newly formed hematoma membrane (1). It predominantly affects elderly individuals, typically following minor trauma, with most cases showing no underlying brain parenchymal injury, and clinical manifestations usually develop over weeks to months (2). With the trend of population aging and increased use of antithrombotic medications among the elderly, the incidence of CSDH has risen (3); studies have predicted that by 2030, its incidence will surpass that of brain tumors to become the most common neurosurgical disease (4). CSDH is characterized by severe symptoms and high recurrence rates, with common manifestations including headache, vomiting, motor deficits, altered consciousness, and even cerebral herniation (5). Data from the Japanese Neurosurgical Database showed that CSDH surgery was the most common neurosurgical procedure in 2018 and 2019 (19.4–18.9%) (6). Its economic impact is substantial, imposing significant financial, physical, and psychological burdens on patients.

The pathogenesis of CSDH remains incompletely elucidated. Studies suggest that head trauma serves merely as a precipitating factor, while formation and progression involve the following processes: local coagulation disorders and repeated hemorrhage from the hematoma membrane (7). Inflammatory reactions (resulting from bridging vein injury caused by minor brain trauma), abnormal angiogenesis, and hyperfibrinolysis play critical roles in disease development (3). The hematoma forms from blood and plasma derived from injured bridging veins, ruptured dural vascular plexuses, microvascular leakage, cerebrospinal fluid, or protein-rich blood cysts (8, 9). Recent studies have demonstrated that local inflammatory reactions play an important role in CSDH formation and progression. Inflammatory cells including neutrophils, lymphocytes, monocytes, and eosinophils have been observed in both the outer membrane and intracavitary fluid of the hematoma, with their presence correlating with recurrence rates, indicating that hematoma formation and development are inseparable from inflammatory responses (10, 11). However, the relationship between disease evolution and peripheral blood inflammatory responses remains unclear.

Current studies have shown that peripheral blood inflammatory markers and their various ratios have been associated with disease severity and outcomes in multiple acute and chronic surgical and non-surgical conditions (12, 13). Several indices and ratios have been investigated to understand their roles in CSDH pathogenesis and prognosis: for example, cell counts, neutrophil-to-lymphocyte ratio (NLR), and platelet-to-lymphocyte ratio (PLR) have been found to be associated with CSDH recurrence (14, 15). Currently, NPAR, TyG, and SII have been confirmed to be associated with stroke, cardiovascular disease, and metabolic-inflammatory diseases (16–18), and are emerging as novel inflammatory markers in routine blood testing. However, their association with CSDH remains to be investigated, which constitutes the objective of this study. We retrospectively analyzed inflammatory cell characteristics in a cohort of CSDH patients to confirm whether CSDH induces a systemic inflammatory response.

## 2 Materials and methods

### 2.1 Research subjects

Between 2023 and 2025, 101 patients admitted to the Department of Neurosurgery, The First Affiliated Hospital of Bengbu Medical University, were screened, and 87 eligible patients were enrolled. CSDH diagnosis was established based on a comprehensive assessment of imaging data, clinical symptoms and signs, and head trauma history. Healthy controls were randomly recruited from the physical examination center according to age and sex proportions of the patients. A total of 130 healthy controls were recruited. Data were extracted from the hospital electronic medical record system; all laboratory examinations were routine admission items, with excellent data completeness. Verification confirmed no missing values across all variables for the 217 enrolled subjects.

Inclusion criteria: (a) Age ≥18 years; (b) No severe trauma, inflammatory disease, surgical treatment, or fever within 3 months; (c) No other central nervous system lesions except CSDH; (d) Clinically diagnosed CSDH.

Exclusion criteria: (a) Psychiatric disorders; (b) Major surgical history prior to this study; (c) Contraindications to medication use; (d) Significant organ organic lesions; (e) Use of glucocorticoids or anti-inflammatory drugs within 3 months (glucocorticoids such as prednisone, methylprednisolone, dexamethasone; non-steroidal anti-inflammatory drugs [NSAIDs] such as aspirin, ibuprofen, diclofenac); (f) Pregnant or postpartum women, history of inflammation or infectious diseases; (g) Patients with incomplete clinical records.

All serum samples were collected from the antecubital vein on the day of hospital admission. The inclusion criteria explicitly required that patients had not experienced severe trauma, undergone surgery for inflammatory diseases, or had a fever within the past 3 months, and had not taken glucocorticoids or anti-inflammatory drugs. All participants provided informed consent by telephone or in writing; for the retrospective portion of the study, the ethics committee approved an exemption from informed consent. All study protocols were approved by the Review Committee of the First Affiliated Hospital of Bengbu Medical University. This study adhered to the principles of the Declaration of Helsinki.

### 2.2 Methods

#### 2.2.1 General data collection

Baseline information of all CSDH patients was retrospectively collected: age, sex, medical history (hypertension, diabetes, cerebral infarction, coronary heart disease, medication history), smoking history. Blood routine and biochemical tests were collected upon admission. All serological samples were collected from the elbow vein on the day of admission and tested in a sterile environment strictly according to kit instructions. Comprehensive serological marker calculation formulas were as follows: NPAR = (neutrophil percentage × 100) / albumin; SII = neutrophil count × platelet count / lymphocyte count; TyG = ln(TG × FBG / 2). Relevant baseline information and laboratory examination data of healthy controls were simultaneously collected. Medication history was defined as at least 1 month of anticoagulant or statin use. This study has the limitation of baseline imbalance in age and sex; subsequent analyses evaluated association robustness through stratification and progressive modeling.

#### 2.2.2 Statistical analysis

Statistical analyses were performed using SPSS 27.0, GraphPad Prism 10.0, and R 4.3.0. Measurement data were tested for normality; normally distributed data were expressed as mean ± standard deviation (Mean ± SD) and compared between groups using independent samples *t*-test; non-normally distributed data were described as median (M) and interquartile range (IQR) and compared using Mann-Whitney U test. Count data were presented as frequency (*n*) and proportion (%), with between-group comparisons using Fisher's exact probability method. Continuous variables (age, NPAR, lipids, blood routine indicators, etc.) were entered into models as raw values; dichotomous variables (sex, hypertension, diabetes, cerebral infarction, coronary heart disease, anticoagulation history, statin medication history) were included as dummy variables, with female or “no” as the reference group. Verification confirmed no missing values across all 217 enrolled subjects.

Univariate logistic regression analysis was first performed to assess the association between baseline characteristics and inflammatory indicators with CSDH. Variables with *P* < 0.05 and clinically recognized confounders (age, sex, hypertension) were included in the multivariate model. Backward stepwise elimination was applied with a removal criterion of *P* > 0.05 until all retained variables were significant. The final model included 6 covariates (NPAR, age, sex, HDL-C, LDL-C, SII), based on 87 CSDH events, yielding an events-per-variable (EPV) ratio of 14.5:1, satisfying the empirical lower limit (10:1). Variance inflation factor (VIF) was used to assess collinearity, with VIF > 5 indicating severe collinearity. Bootstrap resampling (1,000 iterations) was used for internal validation of AUC and regression coefficients, reporting bootstrap means, standard errors, and 95% confidence intervals. Calibration plots were generated to assess consistency between predicted and observed probabilities. The Hosmer-Lemeshow test was used to evaluate model goodness-of-fit, noting that this test has limited statistical power in small samples. Receiver operating characteristic (ROC) curves were plotted, and area under the curve (AUC), optimal cutoff value (at maximum Youden index), 95% confidence interval, sensitivity, and specificity were calculated.

## 3 Results

### 3.1 Characteristics of enrolled patients and controls

**Figure 1:**
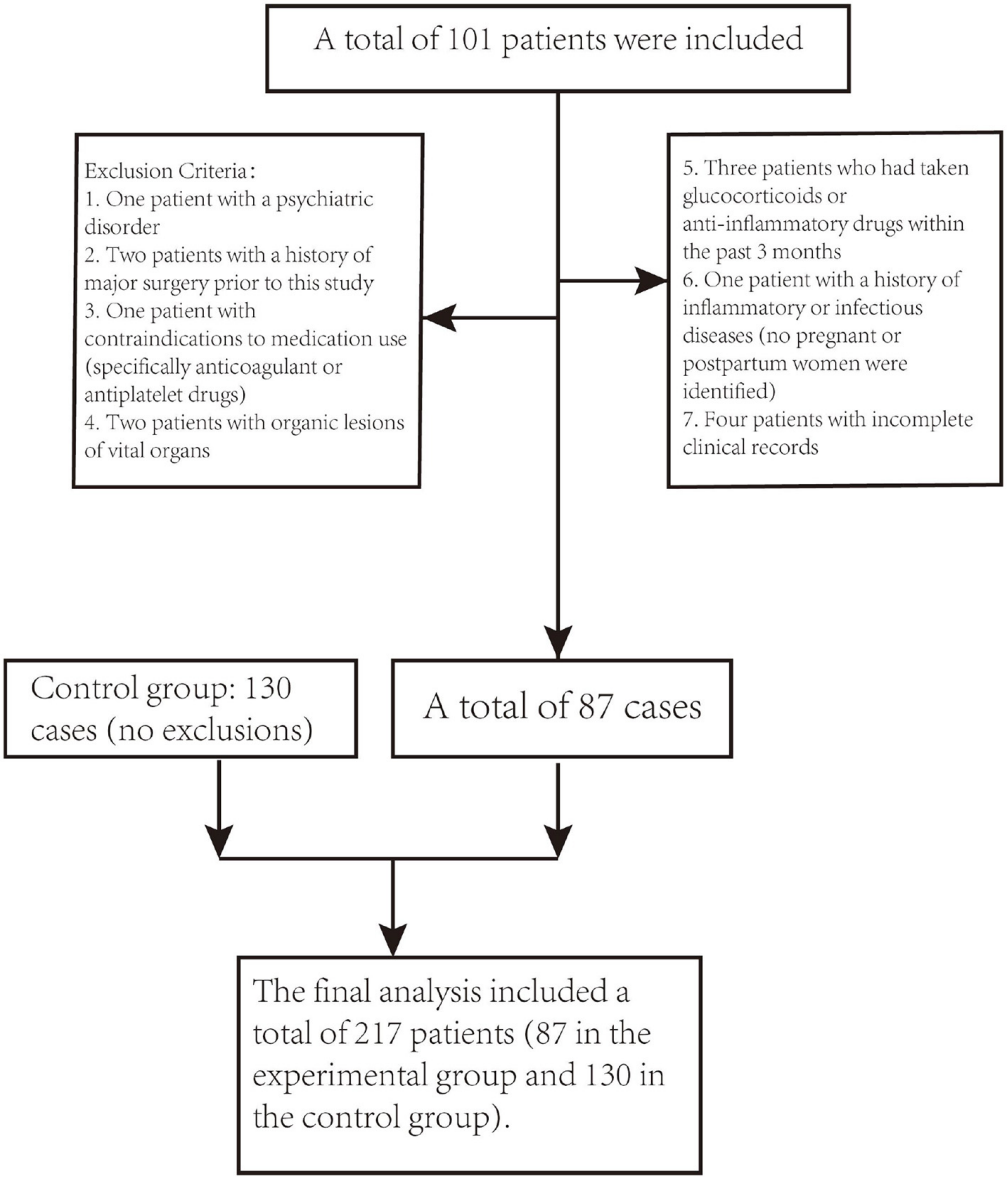
Screening flow diagram.

A total of 217 subjects were enrolled, including 130 controls. Among 101 screened patients, 14 were excluded according to inclusion and exclusion criteria, with 87 patients finally enrolled. The screening process is shown in Figure 1. The mean age of CSDH patients was 70.07 ± 12.32 years, compared to 61.06 ± 9.37 years for controls. Significant differences were observed between groups in age, sex, platelet count, albumin, total cholesterol, HDL-C, NPAR, neutrophil count, lymphocyte count, monocyte count, glucose, urea, total cholesterol, LDL-C, SII, and TyG (*P* < 0.05), while white blood cell count, creatinine, underlying medical history, and medication history showed no significant differences (*P* > 0.05) See Table 1.

**Table 1:** Baseline characteristics.

Variables	Total (*n* = 217)	Control (*n* = 130)	CSDH (*n* = 87)	Statistic	*P*
Age	64.67 ± 11.51	61.06 ± 9.37	70.07 ± 12.32	*t* = −5.79	< 0.001
Sex (%)				χ^2^ = 36.77	< 0.001
Male	134 (61.75)	59 (45.38)	75 (86.21)		
Female	83 (38.25)	71 (54.62)	12 (13.79)		
Coronary artery disease (%)				χ^2^ = 0.50	0.479
No	204 (94.01)	121 (93.08)	83 (95.40)		
Yes	13 (5.99)	9 (6.92)	4 (4.60)		
Hypertension (%)				χ^2^ = 0.39	0.530
No	72 (33.18)	41 (31.54)	31 (35.63)		
Yes	145 (66.82)	89 (68.46)	56 (64.37)		
Cerebral infarction (%)				χ^2^ = 0.86	0.355
No	129 (59.45)	74 (56.92)	55 (63.22)		
Yes	88 (40.55)	56 (43.08)	32 (36.78)		
Diabetes (%)				χ^2^ = 2.71	0.100
No	202 (93.09)	118 (90.77)	84 (96.55)		
Yes	15 (6.91)	12 (9.23)	3 (3.45)		
History of anticoagulant use (%)				χ^2^ = 2.73	0.098
No	78 (35.94)	41 (31.54)	37 (42.53)		
Yes	139 (64.06)	89 (68.46)	50 (57.47)		
History of statin use (%)				χ^2^ = 0.26	0.613
No	189 (87.10)	112 (86.15)	77 (88.51)		
Yes	28 (12.90)	18 (13.85)	10 (11.49)		
White blood cell count 10∧^9^/L	5.86 (5.03, 6.89)	5.79 (5.00, 6.72)	6.04 (5.04, 7.13)	Z = −1.45	0.148
Neutrophil count10∧^9^/L	3.31 (2.70, 4.17)	3.23 (2.66, 3.84)	3.71 (2.94, 4.53)	Z = −3.11	0.002
Lymphocyte count 10∧^9^/L	1.77 (1.44, 2.12)	1.87 (1.57, 2.29)	1.59 (1.27, 1.98)	Z = −4.39	< 0.001
Monocyte count 10∧^9^/L	0.44 (0.35, 0.53)	0.43 (0.32, 0.52)	0.48 (0.39, 0.55)	Z = −1.98	0.048
Platelet count g/L	219.20 ± 61.55	233.35 ± 53.91	198.06 ± 66.34	t = 4.30	< 0.001
Albumin g/L	43.00 ± 3.78	44.81 ± 2.72	40.29 ± 3.51	*t* = 10.14	< 0.001
Glucose mmol/L	4.96 (4.48, 5.36)	5.03 (4.68, 5.51)	4.70 (4.25, 5.22)	Z = −3.66	< 0.001
Creatinine μmol/L	67.00 (56.00, 79.00)	65.50 (55.25, 77.00)	68.00 (60.50, 79.00)	Z = −1.50	0.134
Urea mmol/L	5.20 (4.26, 6.30)	5.01 (4.21, 5.90)	5.60 (4.40, 6.90)	Z = −2.30	0.021
Total cholesterol mmol/L	4.46 ± 1.13	4.97 ± 0.99	3.71± 0.88	*t* = 9.61	< 0.001
Triglycerides mmol/L	1.110 (0.860, 1.570)	1.190 (0.912, 1.610)	0.990 (0.745, 1.255)	Z = −3.129	0.002
High–density lipoprotein mmol/L	1.389 ± 0.380	1.520 ± 0.355	1.193 ± 0.329	*t* = 6.852	< 0.001
Low-density lipoprotein mmol/L	2.440 (1.880, 3.160)	2.900 (2.373, 3.370)	1.940 (1.450, 2.310)	Z = −7.492	< 0.001
SII	394.369 (274.858, 547.814)	372.843 (265.248, 509.558)	451.198 (310.300, 625.317)	Z = −2.265	0.024
NPAR	13.61 ± 2.68	12.47 ± 1.97	15.31 ± 2.71	*t* = −8.38	< 0.001
TyG	8.40 (8.07, 8.75)	8.51 (8.20, 8.88)	8.25 (7.90, 8.49)	Z = −4.10	< 0.001

NPAR, (percentage of neutrophils × 100) / albumin; SII, (neutrophil count × platelet count) / lymphocyte count; TyG, ln(TG × FBG / 2).

### 3.2 Logistic regression analysis of related influencing factors

Univariate logistic regression analysis was performed on the included variables. Results showed that age, sex, neutrophil count, lymphocyte count, platelet count, albumin, glucose, urea, total cholesterol, HDL-C, LDL-C, NPAR, SII, and TyG were statistically associated with CSDH (*P* < 0.05) (Table 2). Given that NPAR, TyG, and SII are composite indicators with mathematical correlations to their original components, to avoid collinearity and over-adjustment, only composite indicators were included in the multivariate model, with original components excluded. Variables with *P* < 0.05 in univariate analysis and clinically recognized confounders (age, sex, hypertension, diabetes, cerebral infarction, coronary heart disease) were included in multivariate analysis using backward stepwise elimination with a removal criterion of *P* > 0.05. Collinearity diagnosis showed VIF values ranging from 1.078 to 3.716 (all < 5), with tolerance values ranging from 0.269 to 0.928 (all > 0.2), indicating no severe collinearity among independent variables. The Hosmer-Lemeshow test χ^2^ = 14.417, df = 8, *P* = 0.072, indicating acceptable model fit, but noting that this test has limited power in small samples, results should be interpreted cautiously. TyG was significant in univariate analysis (OR = 0.38, *P* < 0.001) but was eliminated in the multivariate model, possibly due to collinearity with NPAR, age, and lipids—TyG contains triglycerides and glucose, while NPAR contains albumin, both reflecting metabolic status. To avoid collinearity, only NPAR with superior discriminative efficacy was retained in the multivariate model. HDL-C and LDL-C remained significant in the multivariate model with VIF < 5, suggesting complementary information with NPAR.

**Table 2:** Univariate logistic regression analysis.

Variables	β	S.E	Z	*P*	OR (95% CI)
Age	0.08	0.02	5.28	< 0.001	1.08 (1.05 ~ 1.12)
Sex (%)					
Male					1.00 (reference)
Female	2.02	0.36	5.65	< 0.001	7.52 (3.73 ~ 15.15)
Coronary artery disease (%)					
No					1.00 (reference)
Yes	−0.18	0.29	−0.63	0.530	0.83 (0.47 ~ 1.48)
Hypertension (%)					
No					1.00 (reference)
Yes	−1.05	0.66	−1.58	0.113	0.35 (0.10 ~ 1.28)
Cerebral infarction (%)					
No					1.00 (reference)
Yes	−0.26	0.28	−0.92	0.355	0.77 (0.44 ~ 1.34)
Diabetes (%)					
No					1.00 (reference)
Yes	−0.43	0.62	−0.70	0.482	0.65 (0.19 ~ 2.17)
History of anticoagulant use (%)					
No					1.00 (reference)
Yes	−0.47	0.29	−1.65	0.099	0.62 (0.35 ~ 1.09)
History of statin use (%)					
No					1.00 (reference)
Yes	−0.21	0.42	−0.51	0.613	0.81 (0.35 ~ 1.85)
White blood cell count 10∧^9^/L	0.13	0.08	1.51	0.130	1.13 (0.96 ~ 1.33)
Neutrophil count10∧^9^/L	0.32	0.11	2.85	0.004	1.37 (1.10 ~ 1.71)
Lymphocyte count 10∧^9^/L	−1.25	0.30	−4.18	< 0.001	0.29 (0.16 ~ 0.51)
Monocyte count 10∧^9^/L	1.08	0.81	1.34	0.180	2.96 (0.61 ~ 14.44)
Platelet count g/L	−0.01	0.00	−3.99	< 0.001	0.99 (0.98 ~ 0.99)
Albumin g/L	−0.56	0.08	−6.83	< 0.001	0.57 (0.49 ~ 0.67)
Glucose mmol/L	−0.42	0.15	−2.73	0.006	0.66 (0.49 ~ 0.89)
Creatinine μmol/L	0.01	0.01	1.67	0.096	1.01 (1.00 ~ 1.03)
Urea mmol/L	0.19	0.08	2.30	0.022	1.21 (1.03 ~ 1.42)
Total cholesterol mmol/L	−1.46	0.21	−6.92	< 0.001	0.23 (0.15 ~ 0.35)
Triglycerides mmol/L	−0.21	0.19	−1.14	0.255	0.81 (0.56 ~ 1.17)
High–density lipoprotein mmol/L	−3.07	0.53	−5.78	< 0.001	0.05 (0.02 ~ 0.13)
Low–density lipoprotein mmol/L	−1.58	0.24	−6.52	< 0.001	0.21 (0.13 ~ 0.33)
SII	0.01	0.00	2.64	0.008	1.01 (1.01 ~ 1.01)
NPAR	0.57	0.08	6.72	< 0.001	1.77 (1.50 ~ 2.09)
TyG	−0.97	0.29	−3.41	< 0.001	0.38 (0.22 ~ 0.66)

NPAR, (percentage of neutrophils × 100) / albumin; SII, (neutrophil count × platelet count) / lymphocyte count; TyG, ln(TG × FBG / 2).

Multivariate analysis results showed that NPAR, age, sex, HDL-C, and LDL-C were independently associated with CSDH (*P* < 0.05). Age and NPAR showed positive associations; for sex, with female as reference, male OR = 3.24 (95% CI: 1.27–8.28, *P* = 0.014), indicating higher CSDH risk in males, consistent with the higher male proportion (86.2%) in the overall sample, possibly reflecting selection bias; HDL-C and LDL-C showed negative associations, but given the cross-sectional design, causal inference is not possible. SII OR was 0.99 (*P* = 0.046) with negligible effect size and AUC of only 0.591, lacking clinical discriminative value (Table 3).

**Table 3:** Multivariate logistic regression analysis.

Variables	β	S.E	Z	*P*	OR (95%CI)
Sex (%)					
Female					1.00 (reference)
Male	1.18	0.48	2.46	0.014	3.24 (1.27 ~ 8.28)
Age	0.04	0.02	2.13	0.033	1.04 (1.01 ~ 1.08)
High–density lipoprotein mmol/L	−1.49	0.63	−2.37	0.018	0.23 (0.07 ~ 0.77)
Low-density lipoprotein mmol/L	−0.96	0.30	−3.20	0.001	0.38 (0.21 ~ 0.69)
SII	−0.01	0.00	−2.00	0.046	0.99 (0.99 ~ 0.99)
NPAR	0.63	0.15	4.31	< 0.001	1.88 (1.41 ~ 2.51)

NPAR, (percentage of neutrophils × 100) / albumin; SII, (neutrophil count × platelet count) / lymphocyte count.

### 3.3 ROC curve analysis of different indicators associated with chronic subdural hematoma

**Figure 2:**
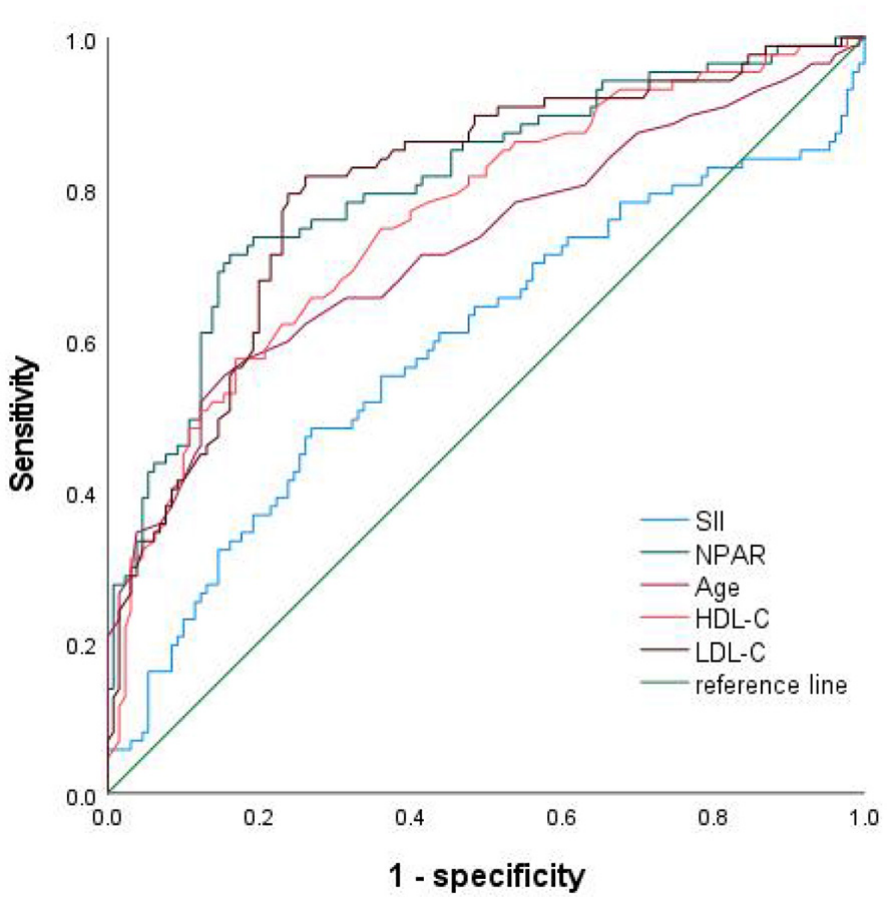
ROC curves of different indicators associated with chronic subdural hematoma.

ROC curves were plotted for NPAR, age, HDL-C, and LDL-C to evaluate the diagnostic specificity of related factors. Sex, as a dichotomous variable, was used for ROC comparison of baseline discriminative efficacy (AUC = 0.704) without clinical cutoff optimization significance, and therefore no curve was plotted. Results showed: ROC curve analysis demonstrated that NPAR exhibited optimal discriminative efficacy for CSDH (AUC = 0.808, 95% CI: 0.75–0.87), with a cutoff value of 14.18, Youden index of 0.551, sensitivity of 71.3%, and specificity of 83.8%. LDL-C ranked second (AUC = 0.800) but showed a negative association, with elevated risk when LDL-C ≤ 2.355 mmol/L; HDL-C (AUC = 0.760) and age (AUC = 0.724) showed moderate efficacy. SII AUC was only 0.591, suggesting limited value as a standalone diagnostic tool See Table 4 and Figure 2.

**Table 4:** The association value of relevant risk factors for chronic subdural hematoma.

Variable	AUC	Optimal cut-off value	Youden index	Sensitivity	Specificity	*P*-value	95% CI
Age	0.724	70.50	0.398	55.2%	84.6%	< 0.001	0.65–0.80
High-density lipoprotein mmol/L	0.760	1.235	0.406	57.5%	83.1%	< 0.001	0.69–0.83
Low-density lipoprotein mmol/L	0.800	2.355	0.555	79.3%	76.2%	< 0.001	0.74–0.86
SII	0.591	498.232	0.214	48.3%	73.1%	0.023	0.51–0.67
NPAR	0.808	14.181	0.551	71.3%	83.8%	< 0.001	0.75–0.87

NPAR, (percentage of neutrophils × 100) / albumin; SII, (neutrophil count × platelet count) / lymphocyte count.

### 3.4 Multivariate model validation

**Figure 3:**
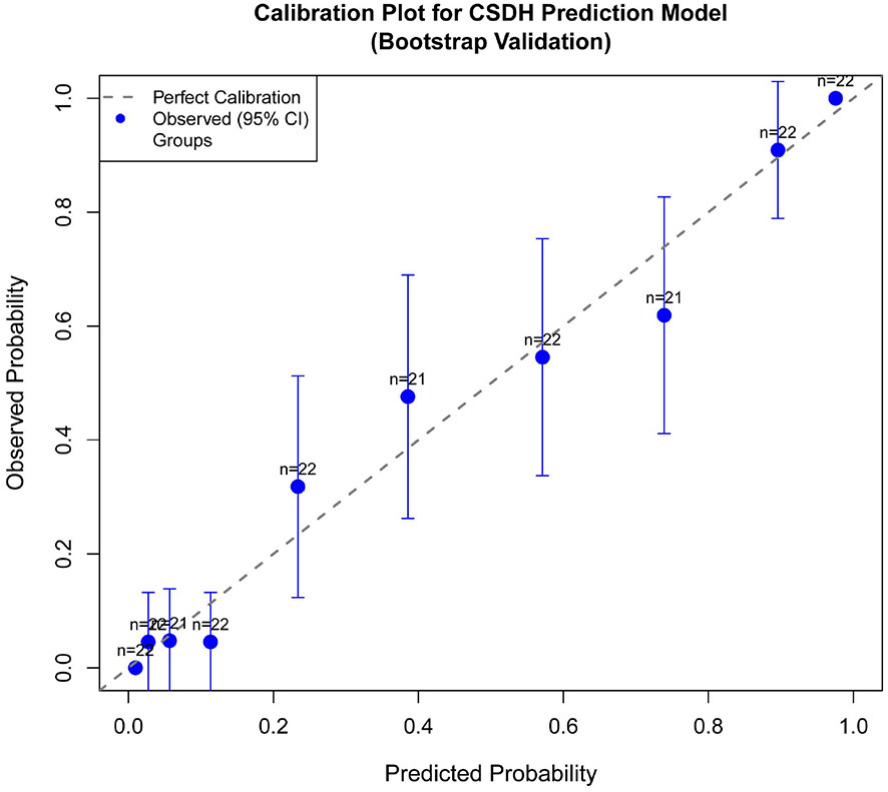
Calibration plot of the multivariate model.

The multivariate logistic regression model demonstrated superior comprehensive discriminative efficacy compared to single indicators. Bootstrap validation showed an AUC of 0.914 (95% CI: 0.882–0.953), with optimism-corrected AUC of 0.907 (Table 5). This AUC reflects the comprehensive predictive ability of NPAR combined with age, sex, and lipids, rather than NPAR's standalone discriminative efficacy. The calibration plot (Figure 3) showed good calibration within the predicted probability range of 0.2–0.8, but slight deviation at extreme probabilities due to sparse samples. The Hosmer-Lemeshow test χ^2^ = 14.417, df = 8, *P* = 0.072, indicating acceptable model fit, but noting limited power of this test in small samples, results should be interpreted in conjunction with calibration plots and bootstrap validation See Table 6.

**Table 5:** Bootstrap validation results.

Indicator	Original value	Bootstrap mean	Standard error	95% CI	Optimism	Corrected value
AUC	0.914	0.920	0.019	0.882–0.953	0.007	0.907

**Table 6:** Bootstrap validation of regression coefficients.

Variable	Original coefficient	Bootstrap mean	Standard error	95% CI	Original OR	OR 95% CI
NPAR	0.632	0.69	0.182	0.361–1.090	1.881	1.435–2.975
Age	0.042	0.045	0.025	0.002–0.097	1.043	1.002–1.102
Sex (male)	−1.176	−1.219	0.554	−2.272~-0.208	0.309	0.103–0.812
HDL-C	−1.488	−1.747	0.816	−3.668~-0.421	0.226	0.026–0.657
LDL-C	−0.957	−1.033	0.371	−1.829~-0.390	0.384	0.161–0.677
SII	−0.002	−0.002	0.001	−0.006~0.000	0.998	0.994–1.000

NPAR, (percentage of neutrophils × 100) / albumin; SII, (neutrophil count × platelet count) / lymphocyte count.

### 3.5 Independent effects of age and sex on the NPAR-CSDH association

Given that this study showed baseline differences between the CSDH group and controls in age (70 vs. 61 years) and sex composition (86% vs. 45% male), and that both factors are known to independently affect neutrophil percentage and serum albumin levels, we used progressive logistic regression models to evaluate the robustness of the NPAR-CSDH association. As shown in Table 7, in the crude model without any covariate adjustment, NPAR was significantly associated with CSDH (OR = 1.768, 95% CI: 1.497–2.087, *P* < 0.05). After adjustment for age and sex only, OR decreased to 1.605 (95% CI: 1.330–1.937, *P* < 0.05), a reduction of approximately 9.6%, suggesting that age and sex explained only a small proportion of the association, with the core association remaining significant. After further inclusion of hypertension, diabetes, cerebral infarction, coronary heart disease, anticoagulation history, statin medication history, and laboratory indicators including glucose, creatinine, urea, total cholesterol, triglycerides, HDL-C, and LDL-C, NPAR remained independently and significantly associated with CSDH (OR = 1.626, 95% CI: 1.238–2.135, *P* < 0.05), with all model VIF values < 5, indicating no severe multicollinearity. These results indicate that the NPAR-CSDH association is not driven by age and sex differences and possesses relative robustness.

**Table 7:** Progressive logistic regression models.

Variables	Model 1		Model 2		Model 3	
	OR (95% CI)	*P*	OR (95% CI)	*P*	OR (95% CI)	*P*
NPAR	1.768 (1.497 ~ 2.087)	< 0.001	1.605 (1.330 ~ 1.937)	< 0.001	1.626 (1.238 ~ 2.135)	< 0.001
Model 1: Crude (unadjusted)						
Model 2: Adjusted for sex, age						
Model 3: Adjusted for sex, hypertension, diabetes, cerebral infarction, coronary heart disease, anticoagulation history, statin medication history, age, glucose, creatinine, urea, total cholesterol, triglycerides, HDL-C, LDL-C						

### 3.6 NPAR comparison stratified by sex and age

To evaluate the confounding effects of age and sex on the NPAR-CSDH association, we compared NPAR levels within strata stratified by sex and age (< 70 years vs. ≥70 years) (Table 8). In three age-balanced strata, NPAR differences were all positive (CSDH group higher than controls), with significant differences in the age-balanced male < 70 years stratum (14.31 ± 1.96 vs. 12.67 ± 2.11, *P* < 0.001) and female ≥70 years stratum (16.30 ± 3.58 vs. 12.71 ± 1.65, *P* = 0.019). The female < 70 years stratum did not reach significance (*P* = 0.360) due to extremely small CSDH sample size (*n* = 3), but the effect direction was consistent. The male ≥70 years stratum showed age imbalance within the stratum (CSDH group 79.5 ± 6.7 years vs. control group 74.2 ± 3.0 years), and results may be affected by residual confounding.

**Table 8:** NPAR comparison stratified by sex and age.

Sex	Male	Male	Female	Female
Age stratum	< 70 years	≥70 years	< 70 years	≥70 years
CSDH group (*n*)	34	41	3	9
CSDH age	58.5 ± 7.5	79.5 ± 6.7	56.3 ± 11.7	75.2 ± 3.4
CSDH NPAR	14.31 ± 1.96	16.03 ± 2.83	13.80 ± 2.29	16.30 ± 3.58
Control group (*n*)	46	13	60	11
Control age	59.5 ± 5.5	74.2 ± 3.0	57.0 ± 8.8	74.2 ± 3.5
Control NPAR	12.67 ± 2.11	12.61 ± 1.69	12.25 ± 1.99	12.71 ± 1.65
Age difference	−1	5.4	−0.7	1
NPAR difference	1.64	3.42	1.55	3.59
*t*-value	3.563	5.327	1.155	2.769
*P*-value	< 0.001	< 0.001	0.360	0.019

NPAR, (percentage of neutrophils × 100) / albumin.

## 4 Discussion

Chronic subdural hematoma (CSDH) is one of the most common neurosurgical diseases, characterized by pathological accumulation of blood in the subdural space with insidious onset and progression (19). Given the increasing incidence among the elderly population, the disease burden is rising with the demographic transition toward aging (20). The pathophysiological cycle of CSDH formation and expansion involves traumatic and inflammatory components, promoting the formation of permeable neovascular membranes (19). Inflammatory cytokines are elevated in the hematoma cavity; these inflammatory factors progressively expand the hematoma by increasing vascular permeability, promoting capillary growth, enhancing fibrinolytic activity, and recruiting immune cells (21, 22). The lack of improvement in treatment outcomes over the past decades has made this disease a focus of pathophysiological and novel therapeutic research. This study preliminarily explored the association of NPAR, an inflammation-nutrition composite indicator, in primary CSDH, providing a complementary perspective to previous studies focused on single inflammatory markers. Compared with traditional risk factors (age, sex) and single inflammatory indicators (SII), NPAR, as a composite inflammation-nutrition marker, provides a new serological tool for early identification of CSDH while maintaining high specificity (83.8%), with a reference cutoff value (≥14.18) for statistical association.

This study reports AUC results at two levels: in univariate analysis, NPAR AUC was 0.808, demonstrating its discriminative efficacy as a single inflammation-nutrition indicator superior to traditional lipid and demographic factors; in the multivariate model, AUC increased to 0.914 after combining NPAR with age, sex, and lipids, suggesting that comprehensive prediction may further improve accuracy. However, the clinical operability of the multivariate model is inferior to that of a single indicator, and bootstrap validation showed unstable coefficient estimates for sex and HDL-C in small samples (wide 95% CIs). Therefore, NPAR as a single indicator (AUC = 0.808) remains the core finding of this study, while the multivariate model (AUC = 0.914) serves as exploratory analysis, suggesting that future comprehensive scoring systems may be developed.

Previous studies have explored the association between various inflammatory markers and CSDH (23), but with certain limitations. Frati et al. (24) focused on local inflammatory mechanisms in postoperative recurrence (IL-6/IL-8), but their core finding—that inflammatory reactions are key drivers of CSDH pathological progression—is highly consistent with our results. Barić et al. (15) found associations between NLR, PLR, and SII with CSDH, but with small sample sizes and no focus on primary risk assessment. Edlmann et al. (21) and Hohenstein et al (25) revealed local inflammatory mechanisms through hematoma cavity cytokine profiling but did not involve systemic inflammation-nutrition indicators. Fan et al. (11) reported elevated peripheral blood neutrophil proportions in CSDH patients but did not evaluate the discriminative efficacy of composite indicators. Compared with these studies, the differences of this study lie in: (1) focusing on primary CSDH rather than recurrence; (2) using the NPAR composite indicator, simultaneously integrating inflammatory (neutrophil percentage) and nutritional (albumin) information, rather than single cell count ratios.

This study performed correlation analyses based on hematological laboratory data collected at patient admission. In recent years, SII, TyG, and NPAR have emerged as simple, novel, and inexpensive indicators widely used to assess inflammatory status. Studies have demonstrated that elevated levels of SII, AISI, and NPAR are more commonly observed in acute and chronic inflammatory diseases, immune disorders, and malignancies (26–28). However, their value in CSDH has not yet been reported. The present study found that NPAR levels were significantly higher in CSDH patients than in controls, and this association remained independent of age, sex, and lipid levels, suggesting that a higher NPAR status may represent an important component in the occurrence and progression of CSDH. Notably, males accounted for 86.2% of CSDH patients in this study, consistent with the male-to-female ratio (approximately 3:1 to 4:1) reported in previous literature (29). In the multivariate model, the risk in males was significantly higher than in females, indicating that elderly males with even mildly elevated NPAR should be regarded as a high-risk population in clinical screening. The potential advantage of NPAR over NLR, PLR, and SII lies in its simultaneous reflection of both inflammatory hyperactivity and nutritional deficiency. NLR and PLR reflect only leukocyte subset proportions, while SII incorporates platelet count but neglects nutritional status. In contrast, the albumin component of NPAR can indicate impaired vascular endothelial repair capacity and decreased colloid osmotic pressure associated with hypoalbuminemia, which is more consistent with the common geriatric frailty status observed in CSDH patients.

The pathophysiological basis of elevated NPAR may involve dual mechanisms: inflammatory hyperactivity and malnutrition. On one hand, elevated neutrophil percentage reflects acute or chronic stress states; neutrophil-released elastase (30) and reactive oxygen species can damage vascular endothelium, promoting increased fragility of dural vessels and hematoma expansion (31, 32); on the other hand, low albumin indicates malnutrition or hepatic dysfunction, leading to decreased colloid osmotic pressure and impaired tissue repair capacity (33), consistent with the common geriatric frailty status in CSDH patients.

In this study, LDL-C and HDL-C showed negative correlations with CSDH (OR < 1), but the directionality of this association is difficult to determine. Low cholesterol levels in elderly hospitalized patients may be influenced by multiple non-causal factors, including statin therapy, acute-phase reactions, or dietary changes during hospitalization, rather than purely reflecting malnutrition or inflammatory status. Previous studies have reported associations between HDL-C and CSDH recurrence (32, 33), but pathophysiological mechanisms may differ between recurrence and primary onset; this study employed a cross-sectional design, unable to distinguish whether low cholesterol is a cause or consequence of CSDH, and therefore mechanisms from recurrence literature should not be extrapolated to primary CSDH. Additionally, statin use in this study (12.9%) was lower than expected in the elderly population, possibly reflecting inadequate medication history assessment, but no stratified analysis was performed to verify the effect of statin use on the lipid-CSDH association, representing a limitation of this study. In summary, low cholesterol levels show statistical association with CSDH but cannot be inferred as causal risk factors.

In this study, SII showed *P* = 0.046 in the multivariate model, but OR was only 0.99 with negligible effect size. This “statistically significant but clinically meaningless” result suggests limited value of SII in CSDH prediction; its marginal significance may be due to sample size or measurement units rather than true biological association. Therefore, this study does not recommend SII as a screening or diagnostic indicator for CSDH.

The clinical value of this study lies in providing a low-cost, easily accessible tool for CSDH high-risk population screening. NPAR calculation requires only routine blood routine and biochemical tests, with no additional cost. This study employed a case-control design with healthy physical examination populations as controls, differing from the elderly symptomatic populations requiring differential diagnosis in clinical practice; therefore, NPAR ≥14.18 currently serves only as a reference node for statistical association rather than a clinical diagnostic cutoff. When NPAR ≥14.18, enhanced imaging monitoring is recommended for elderly males with recent minor head trauma history and elevated NPAR.

This study has the following limitations: First, the male ≥70 years stratum showed age imbalance within the stratum due to insufficient elderly control samples (5.3-year difference), and results may be affected by residual confounding; the female < 70 years stratum had extremely small CSDH sample size (*n* = 3) with insufficient statistical power. Second, the cross-sectional nature of the case-control design cannot establish the temporal relationship between NPAR and CSDH; elevated NPAR may be a secondary change during CSDH course (reverse causality). Third, the single-center design limits generalizability of results. Fourth, as a composite inflammation-nutrition indicator, NPAR's two components are influenced by multiple non-CSDH factors (such as infection, hepatic and renal function), with limited specificity; no stratified analysis of statin use was performed to verify the lipid-CSDH association. Finally, traditional inflammatory markers such as CRP and IL-6 were not measured, preventing direct comparison of NPAR's relative advantages.

## 5 Conclusion

This study preliminarily explored the association of NPAR, an inflammation-nutrition composite indicator, in primary CSDH, providing a complementary perspective to previous studies focused on single inflammatory markers. The study found a significant overall association between neutrophil percentage-to-albumin ratio (NPAR) and chronic subdural hematoma (CSDH), which remained significant within age- and sex-matched strata (male < 70 years, female ≥70 years), supporting NPAR's independent association value as a CSDH-related inflammation-nutrition indicator. However, limitations in the male elderly stratum and female young stratum suggest that NPAR should not be used as a single diagnostic criterion but rather as an auxiliary reference for comprehensive risk assessment. Future prospective cohort studies are needed to validate the predictive role of baseline NPAR and to explore the impact of nutritional interventions on NPAR levels and disease prognosis.

## Data Availability

The raw data supporting the conclusions of this article will be made available by the authors, without undue reservation.
